# Management of Tomato Bacterial Canker Disease by the Green Fabricated Silver Nanoparticles

**DOI:** 10.1186/s12870-024-05238-7

**Published:** 2024-06-25

**Authors:** Muhammad Arif Hussain, Aneela Nijabat, Muhammad Mahmood ur Rehman, Rahmatullah Qurashi, Manzer H. Siddiqui, Saud Alamri, Zia -ur-Rehman Mashwani, Saad Ullah Khan Leghari, Muhammad Anwer Shah, Qamar uz Zaman

**Affiliations:** 1 Department of Botany, Pir Mehr Ali Shah Arid Agriculture University, Rawalpindi, Pakistan; 2 Department of Botany, Ghazi University, Dera Ghazi Khan, Pakistan; 3 Department of Botany, University of Mianwali, Mianwali, 42200 Pakistan; 4 School of Agricultural Engineering, Jiangsu University, Zhenjiang, 212013 China; 5 Department of Botany and Microbiology, College of Science, King Saud University, Riyadh, 11451 Saudi Arabia; 6 Department of Environmental Sciences, The University of Lahore, Lahore, 54590 Pakistan

**Keywords:** Canker, Green fabricated, Inoculum, Tomato, Silver nanoparticles

## Abstract

Bacterial canker disease caused by *Clavibacter michiganensis* is a substantial threat to the cultivation of tomatoes, leading to considerable economic losses and global food insecurity. Infection is characterized by white raised lesions on leaves, stem, and fruits with yellow to tan patches between veins, and marginal necrosis. Several agrochemical substances have been reported in previous studies to manage this disease but these were not ecofriendly. Thus present study was designed to control the bacterial canker disease in tomato using green fabricated silver nanoparticles (AgNps). Nanosilver particles (AgNPs) were synthesized utilizing *Moringa oleifera* leaf extract as a reducing and stabilizing agent. Synthesized AgNPs were characterized using UV–visible spectroscopy, scanning electron microscopy (SEM), X-ray diffraction (XRD), energy-dispersive X-ray (EDX), and Fourier transform infrared spectrometry (FTIR). FTIR showed presence of bioactive compounds in green fabricated AgNPs and UV-visible spectroscopy confirmed the surface plasmon resonance (SPR) band in the range of 350 nm to 355 nm. SEM showed the rectangular segments fused together, and XRD confirmed the crystalline nature of the synthesized AgNPs. The presence of metallic silver ions was confirmed by an EDX detector. Different concentrations (10, 20, 30, and 40 ppm) of the green fabricated AgNPs were exogenously applied on tomato before applying an inoculum of *Clavibacter michigensis* to record the bacterial canker disease incidence at different day intervals. The optimal concentration of AgNPs was found to be 30 µg/mg that exhibited the most favorable impact on morphological (shoot length, root length, plant fresh and dry weights, root fresh and dry weights) and physiological parameters (chlorophyll contents, membrane stability index, and relative water content) as well as biochemical parameters (proline, total soluble sugar and catalase activity). These findings indicated a noteworthy reduction in biotic stress through the increase of both enzymatic and non-enzymatic activities by the green fabricated AgNPs. This study marks a first biocompatible approach in assessing the potential of green fabricated AgNPs in enhancing the well-being of tomato plants that affected with bacterial canker and establishing an effective management strategy against *Clavibacter michiganensis*. This is the first study suggests that low concentration of green fabricated nanosilvers (AgNPs) from leaf extract of *Moringa oleifera* against *Clavibacter michiganensis* is a promisingly efficient and eco-friendly alternative approach for management of bacterial canker disease in tomato crop.

## Introduction

Tomato (*Lycopersicon esculentum* L.) from family solanaceae is one of the most nutritive fruit which is widely consumed as fresh or processed vegetables in the world [1]. It is an enriched source of various important contains bioactive compounds including vitamins, nutrients, antioxidants, fibers with less fat contents which add several health benefits [2]. Tomato is 2nd to potato in solanaceous crops in terms of production, consumption and market value [3]. Due to global consumption and formulating various products by the developed countries, there is an increasing trend in the production and cultivation of this crop throughout the world [4]. Global tomato production was 44.2 million metric tons in 2023 with is 5.8 million metric tons higher than annual tomato production in 2022 [5]. According to a survey, California, Italy and China are the major tomato exporter in the international market. Unfortunately, Pakistan stood far below in global tomato production at 32nd position with 0.43% share in international market [6]. This low tomato production in Pakistan can be attributed to several factors including agricultural policies, government policies, extreme weather events (heat, drought, salinity, soil infertility or toxicity), and various fungal, viral and bacterial diseases.

Among bacterial diseases, bacterial canker (BC) caused by *Clavibacter michiganensis* subsp. *michiganensis* causes severe yield losses (ranging from 46 to 93%) in tomato across the world including Pakistan [7, 8]. The BC disease is characterized by white raised lesions on leaves, and stem with yellow to tan patches between veins, and marginal necrosis leading to wilt, stunted growth, small fruit size with brown or white raised lesions [9, 10]. Several chemicals such as copper sulphate, copper hydroxide, streptomycin etc. have been used to reduce the disease incident to some extent, however, the outcomes are not satisfactory and compounds are also not eco-friendly [11]. Thus, there is dire need to introduce novel practical approaches to control the BC disease in tomato and previous studies suggest that use of green fabricated non-materials could be effective control measure for disease management in crops including tomato. Plant parts including bark, root, stem, leaves, fruit and seeds have been found very effective for green fabrication of various nano-materials as they contain the bioactive secondary metabolites of plant extract [12–14]. Several plant extracts have been previously used for green synthesis of various nanomaterials to enhance the crop yield and disease management [15]. Several physic-chemical characters of these nano-materials play key role in their effectiveness like small sized nanomaterials are considered as more efficient in their antimicrobial action in comparison to large sized due to high penetration ability [16].

In the land of tomato agriculture, green fabricated nanoparticles are synthesized by using biomolecules and plant extracts have been found very effective in disease management in several studies [17]. It has been reported that several nanoparticles (zinc, gold, silicon, and silver etc.) kills the microbes by production of reactive oxygen species, lipid peroxidation, DNA and protein degradation, and ATP depletion [18]. Additionally, green fabricated silver nanoparticles have antimicrobial potential and have been very effective in microbial disease management in tomato such as early blight, wilt, and canker diseases. Previous studies suggested silver nanoparticles induce the oxidative damage leading to disruption of several cellular processes including signal transduction and gene expression within pathogenic cells [19] while they have been reported to improve the stress tolerance in plants by enhanced stress responsive enzymatic and non-enzymatic antioxidants activity [20]. Thus, current scenario demands the efficacy of green fabricated AgNps against *Clavibacter michiganensis* subsp. *michiganensis* caused BC disease in tomato. Furthermore, there is dire need to optimize the application technique, concentration and comparative studies in control and open field conditions to evaluate the role of green fabricated AgNPs in tomato crop. Keeping in view the recent positive outcomes, present study was designed to evaluate the role of AgNPs fabricated with leaf extract of *Moringa oleifera* to control and manage the bacterial canker disease in tomato disease (BC).

## Materials and Methods

### Experimental Site and Treatment Plan

Present research work was performed at Ecological Laboratory, Department of Botany, PMAS Arid Agriculture, Pakistan. Two tomato varieties (UJJAWAL and XINFENG) were acquired from NARC Islamabad. Inoculum was prepared from bacterial strain (*Clavibacter michiganensis* subsp. *michiganensis*) to infect both tomato varieties. All healthy plants without infection and without any foliar application were regarded as control whereas, both healthy and infected plants were exposed to various foliar treatments of plant leaf extract and green fabricated AgNPs. All experimental treatments were arranged using completely randomized design with three replications (Table 1).

**Table 1:** Experimental details of study

Variety	Treatment	Concentration
UJJAWAL	T0 (Control)	……
	T0^+^*Moringa* Leaf Extract	5.0 ml
	T0^−^*C. michiganensis inoculum*	……
	T1 *C. michiganensis inoculum* + AgNPs	10 ppm
	T2 *C. michiganensis inoculum* + AgNPs	20 pm
	T3 *C. michiganensis inoculum* + AgNPs	30 ppm
	T4 *C. michiganensis inoculum* + AgNPs	40 ppm
XINFENG	T0 (Control)	……
	T0^+^*Moringa* Leaf Extract	5.0 ml
	T0^−^*C. michiganensis inoculum*	……
	T1 *C. michiganensis inoculum* + AgNPs	10 ppm
	T2 *C. michiganensis inoculum* + AgNPs	20 pm
	T3 *C. michiganensis inoculum* + AgNPs	30 ppm
	T4 *C. michiganensis inoculum* + AgNPs	40 ppm

### Green Synthesis and Characterization of Silver Nanoparticles

#### Preparation of Plant Extract Synthesis of Silver Nanoparticles (AgNPs)

Fresh leaves of *Moringa oleifera* were collected from the garden of the main campus and initially were washed thoroughly with tap water and monitored to replace residues of dirt and other visible particles during drying. Approximately 40 g of dried leaves were placed into the 500 ml beakers in 400 ml sterilized water and boiled for 30 min on a hot plate. Whatman no.1 filter paper was used to filter the extract and filtrate was stored at 4℃. After that, 5mM solution of AgNO_3_ was mixed with plant extract in 1:4 ratios (i.e. 200 ml leaves extract and 800 ml AgNO_3_ solution) in a conical flask. The pH was maintained to 8.0 in the mixture and was kept on hot plate until the solution color did not change into dark brown at room temperature. After that, solution was centrifuged at 10,000 rpm for 15 min and supernatant containing nano-silver particles in 70% ethanol was incubated at 65^o^C for 24 h for drying. Obtained AgNPs were subjected to characterization analysis [21].

### Characterization of Silver Nanoparticles

The green fabricated AgNPs were dissolved in distilled water by sonication for 10 min and were analyzed for UV-Visible spectra and FT-IR spectra using HALODB-20 Spectrophotometer and Fourier Transforms Infrared Spectroscopy (FT-IR). The surface profile of the green fabricated AgNPs was assessed using Scanning Electron Microscope (SEM; SIGMA model as MIRA3; TESCAN Brno and the Czech Republic) at the Institute of Space and Technology (IST), Islamabad. The samples were prepared by merely keeping a lesser quantity of sample on the carbon-coated copper grid for SEM. Then air becomes dry and SEM micrographs were poised at various magnifications. The AgNPs were dried out and drop-coated onto carbon film for elemental analysis. Using an EXD detector, the energy dispersive X-ray (EDX) analysis was implemented.

### Bacterial Strain

**Fig. 1:**
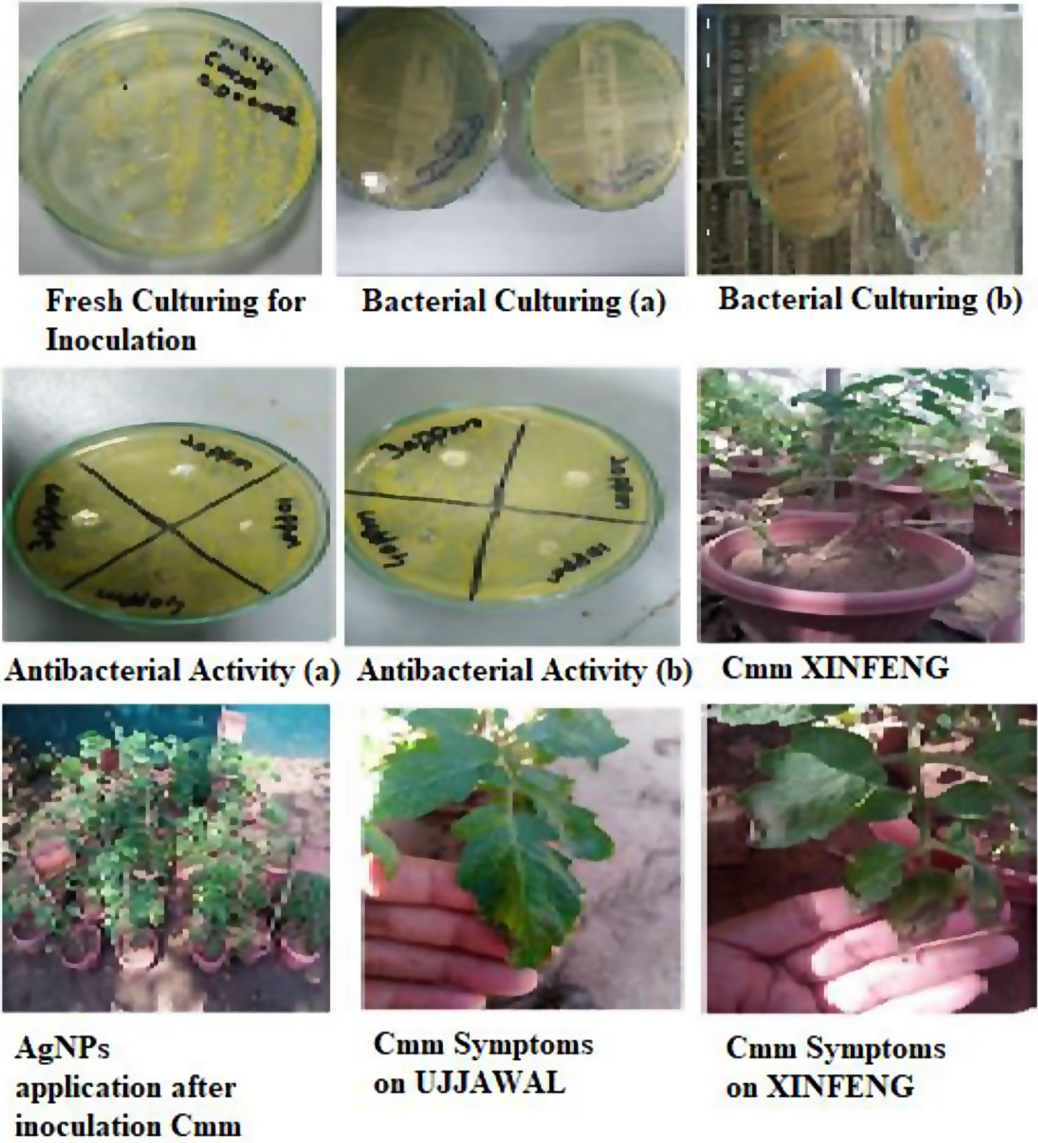
Flow sheet of bacterial culturing and inoculation for tomato plants with AgNPs application

The bacterial strain of *Clavibacter michiganensis* subsp. *michiganensis* (*Cmm*) was selected to induce bacterial canker (BC) and wilt diseases in tomatoes. Inoculum of *Cmm* strain was prepared in nutrient agar (yeast extract; 0.3 g, peptone; 0.5 g, NaCl’ 0.5 g, and agar; 1.5 g) plates at neutral pH 7.0 ± 0.3. All typical effective measures were opted while streaking of solidified media plates to prevent the external contamination. For streaking purposes, purified inoculation loops were used which were tied with paraffin tapes and covered in aluminum foil while with optimum culture conditions, media plates were kept in the growth chamber for 2–3 days because bacteria can be grown up. Single colony was selected and picked from the NA media plate after 2-days cultures and was immersed in 5 ml of liquid media and incubated for overnight at 26-28^o^C. After one day, cultures were centrifuged at 10,000 rpm for 10 min at 25^o^C to isolate a bacterial cel (Fig. 1).

### Crop Husbandry and Disease Inoculation

Study was conducted in medium sized pots (10 × 8-inch) filled with blend of soil, farm yard compost, gravel, and NPK (nitrogen, phosphorus, and potassium) and healthy plants were selected from the nursery and transplanted. These plants were placed in a greenhouse for further growth and were watered daily as a prerequisite. Later on 2 months old plants were infected with bacterial strain *Clavibacter michiganensis* subsp. *Michiganensis* individually (Fig. 1). Total of 15 plants per treatment from both varieties in all replicate were maintained in pots. The three leaves were selected per plant for inoculation. The ad-axial side of leaves was caused by physical injury with needle and inoculated the 1 ml syringes of strain serial dilution in plants.

### Procedure for Data Collection

The data was collected at the 5 days’ time interval. Healthy plants were used as positive control and infected plant with pathogen was used as a negative control. The foliar application of green fabricated silver nanoparticles was initiated after three weeks of applied inoculums and with different concentrations to respective pots at 10 ppm, 20 ppm, 30 ppm, 40 ppm of AgNPs as well as *Moringa oleifera* leaves extract to assess the growth of both tomato varieties at vegetative growth stage. Following observations were recorded:

### Morphological Parameters

Three plants from each treatment were used for root and shoot length measurements. These plants were cleaned with distilled water and roots were detached from shoot and length of shoot and root was recorded in cm (centimeter) using scale. Fresh weight of shoot and root in g was measured separately and then were kept in oven at 65 ^◦^C to determine dry weight in g using digital weight balance.

### Physiological Parameters

For measuring relative water contents, leaf fresh weight was recorded and then the leaf drenched in water for 24 h. After this the leaves saturated weight was recorded. Then these leaves of each sample were kept in oven at 70℃ for one week and after one week the dry weight was recorded [22]. To determine out relative water contents of leaves following equation is used: RWC = Fresh weight–Dry weight) ×100 (Saturated weight–Dry weight). To measure the membrane stability index, the method of Sairam [23] was followed and calculated with following formula: Membrane stability index= [1-C1/C2] ×100.

Leaf chlorophyll contents were measured applying spectrophotometer through after the process of Esra at el [24]. . Following equations was used to calculate chlorophyll contents.

Chlorophyll a contents = 12.7(A663) – 2.7(A645) Chlorophyll b contents = 22.9(A645) – 4.7(A663).

Total chlorophyll contents (A652 × 1000/34.5) A = Absorbance of different wavelengths i.e. (A 663 nm), (A 645 nm), (A652 nm)

### Biochemical Parameter

Proline contents were meashred by using the following formaula:

Total Proline (µg/ml) = (Sample absorbance× Dilution factor × K value) / Fresh weight of plant tissue [24]. While, total soluble sugars were also estimated by following the protocol described by Esra et al. and using the formula:

Sugar (µg/ml) = Sample absorbance × Dilution factor × K value/ Weight of fresh plant tissue Moreover, Catalase activity was measured according to protocol reported by Aebi [25].

### Statistical Analysis

Statistical Package for Social Sciences (SPSS) was used for statistical evaluation of the results. The randomized complete block design (RCBD) was used. All the results were interpreted as mean of S.E from the independent biological replicates.

## Results

### Fabrication and Characterization of Silver Nanoparticle

**Fig. 2:**
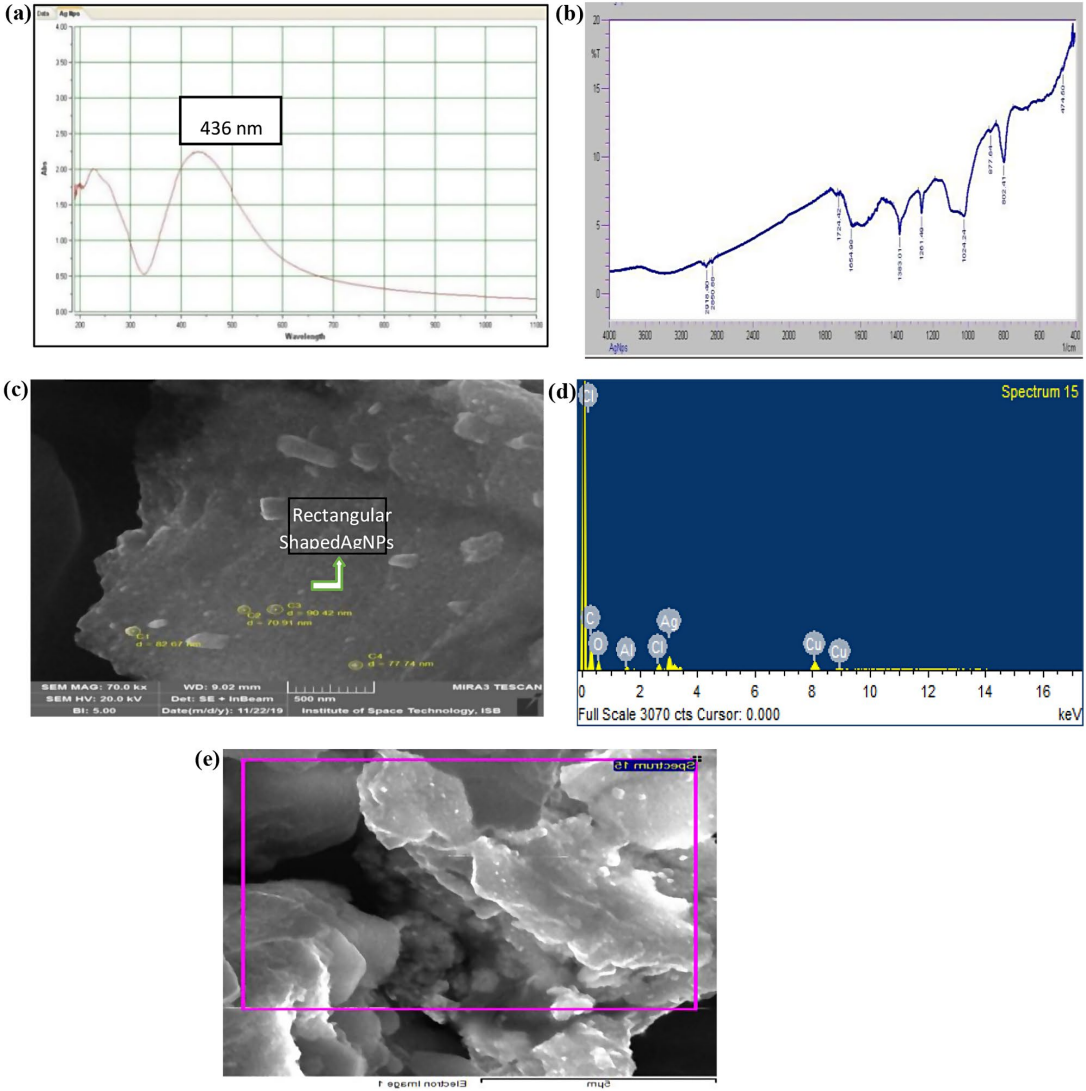
UV-Visible spectrophotometer analysis **(a)**, FT-IR spectrum **(b)**, Scanning electron microscopic micrograph **(c)**, Energy dispersive X-RayEDX **(d)** and Energy dispersive X-Ray EDX Analysis **(e)** of green fabricated AgNPs using extract fresh leaf extract of *Moringa oleifera*

UV-Visible spectra of green fabricated AgNPs showed an extensive peak at 436 nm wavelength and peak value was 2.245 (Fig. 2a). FT-IR spectra (ranging from 440 to 4000 cm^− 1^) of green fabricated AgNPs suggest the presence of biomolecules that could be responsible for reducing and capping of AgNPs in aqueous solution. Total of 10 peaks at 2918.40, 2850.88, 1724.42, 1654.98, 1383.01, 1261.49, 1024.24, 877.64 cm^− 1^ were observed in FT-IR spectrum which could be esters, alcohols, ethers, carbonyl compounds, alkanes, alkenes, alkyl halides, amines, aldehydes and aromatic compounds (Fig. 2b). Presence of NO_2_ was detected at 1383.01 cm^− 1^ from metal precursor solution of AgNO_3_. The AgNPs exhibited two high-pitched absorption peaks at 1654.98 and 2918.40 cm^− 1^ with relation of protein. Due to existence of amide bond potential from carbonyl group of protein biosynthesized AgNPs, an absorption peak was observed at 1724.42 cm^− 1^ whereas, a peak was observed at 2918.40 cm^− 1^ in FT-IR spectrum due to OH group in alcohols and phenols. Confirmation of all these compounds suggest that leaf extract of *Moringa Olifera* can be an appropriate reducing and capping agent for green fabrication of AgNPs in aqueous solution. Rectangular or irregular shape of green fabricated AgNPs having size of 80 to 90 nm was observed in micrographs obtained by scanning electron microscopy (Fig. 2c). The existence of green fabricated AgNPs was furthermore confirmed by energy peak at 3 keV in EDX graphs (Fig. 2d and e).

### Morphological Parameters

**Fig. 3:**
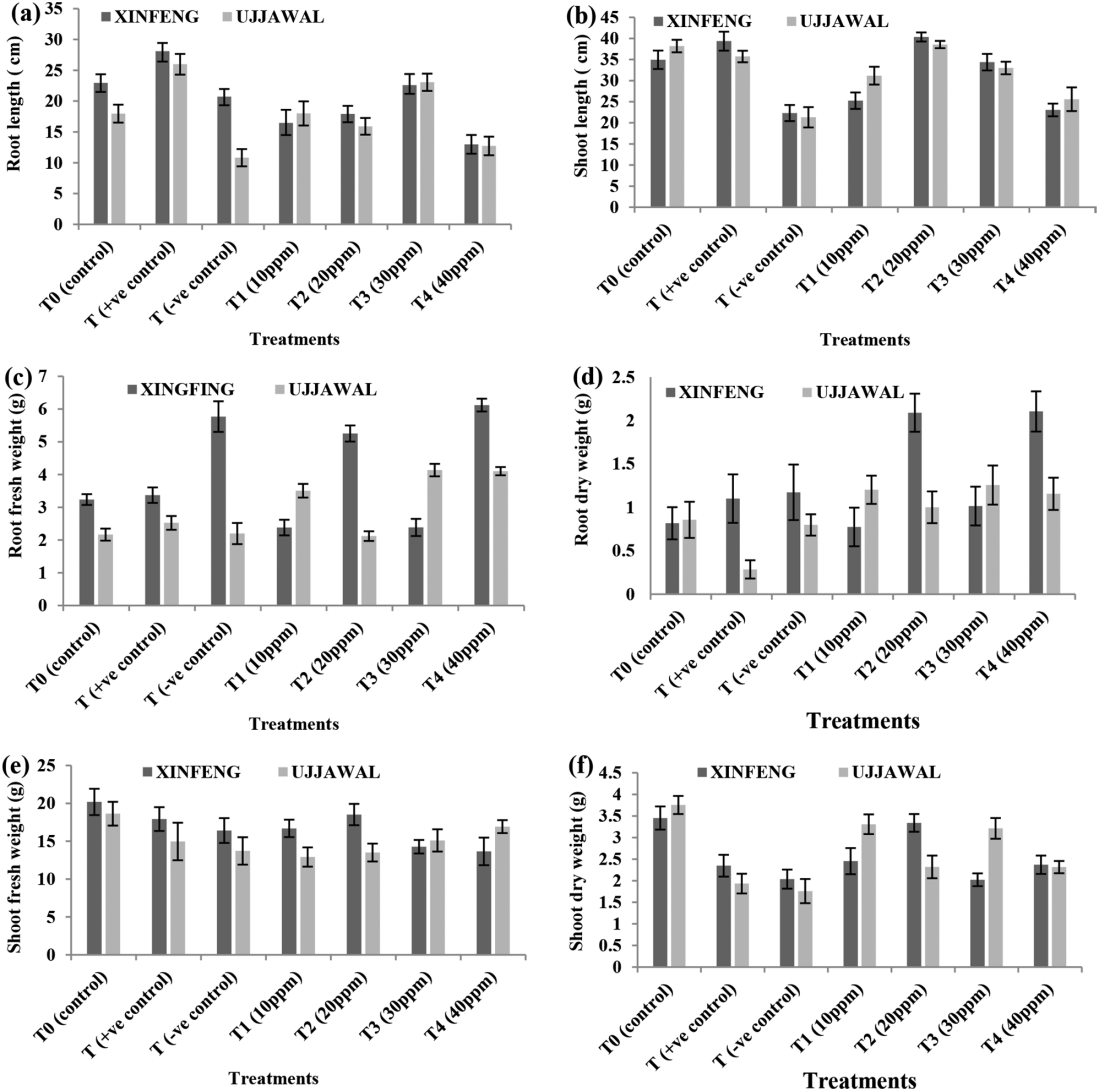
Variation in morphological parameters of two tomato varieties in response of various treatments of green fabricated AgNPs

Significant variation (*p* ≤ 0.05) for morphological parameters of tomato plants of both varieties was observed in response of exogenous application of green fabricated AgNPs. Infected plants of both tomato varieties XINFENG and UJJAWAL from T0^−^ exhibited a marked reduction in root length as compared to healthy plants from T0. On the other hand, Plants treated with leaf extract of *Moringa oleifera* (T0^+^) showed higher root length than plants grown without any treatment (T0) in both varieties of tomatoes. Whereas, optimum root length was recorded for plants treated with foliar application of 30 ppm solution of green fabricated AgNPs (T3) in both varieties among all the treatments, (Fig. 3a). Similar observations were recorded for shoot length of tomato plants of both varieties under control treatments (T0, T0^+^, T0^−^) but maximum shoot length was recorded in response to foliar application of 20 ppm solution of AgNPs (Fig. 3b). Maximum root fresh weight was recorded for plants treated with foliar application of 40 ppm solution of AgNPs (Fig. 3c) while shoot fresh weight was maximum in response of 20 ppm of AgNPs (Fig. 3d) in both varieties. Similar observations were recorded for shoot and root dry weight (Fig. 3e and f). It was concluded from the present study that morphological growth of both selected verities of tomato significantly differ in response of low concentration of green fabricated AgNPs while non-significant was observed in response of foliar application of high concentration of green fabricated AgNPs. Additionally, overall growth rate of XINFENG in terms of morphological traits was comparatively higher than UJJAWAL.

### Physiological Parameters

**Fig. 4:**
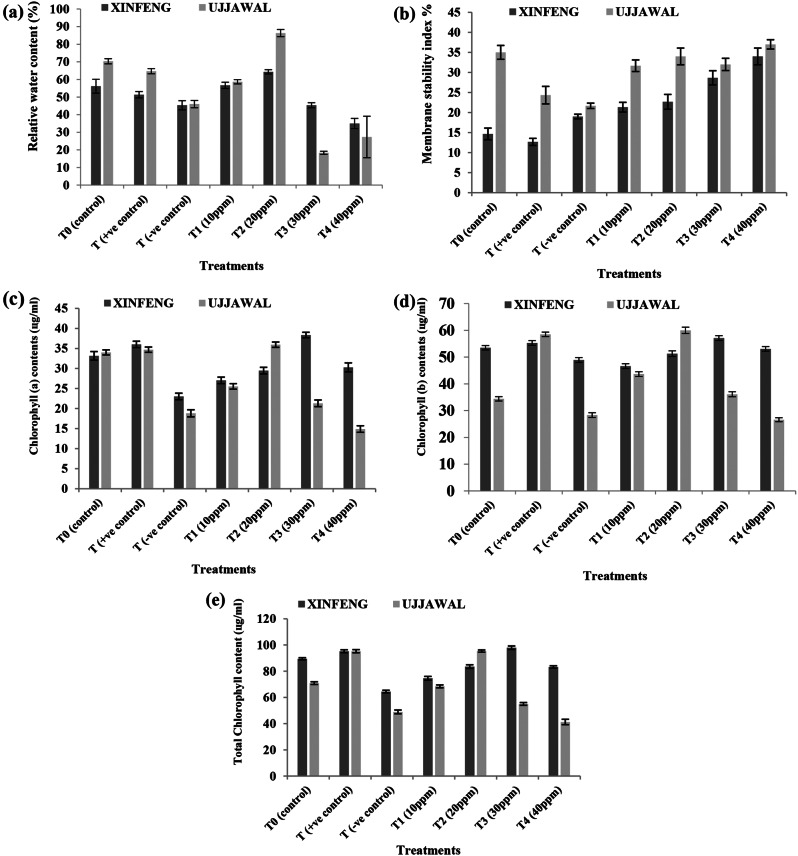
Variation in physiological parameters of two tomato varieties in response of various treatments of green fabricated AgNPs.

Physiological growth of tomato plants was evaluated in terms of relative water contents, membrane stability index, and chlorophyll contents. It was observed in present study that there was a clear decline in relative water contents in response of bacterial canker disease infestation due to inoculation of *Clavibacter michiganensis* (T0^−^) in both varieties as compared to control (T0). The exogenous application of leaf extract of *Moringa oleifera* (T0^+^) and 20 ppm solution of AgNPs (T2) improved the relative water content (Fig. 4a). Similarly, there was significant decrease in membrane stability index in response to disease infestation but surprisingly as the levels of foliar application of green fabricated AgNPs was increased, membrane stability index was also increased and it was highest at 40 ppm (Fig. 4b). Foliar application of AgNPs positively affected the chlorophyll (a), chlorophyll (b), and total chlorophyll contents and maximum values were recorded in response of 30 ppm solution of AgNPs followed by20 ppm solution of AgNPs (Fig. 4c, d, e).

### Biochemical Parameters

**Fig. 5:**
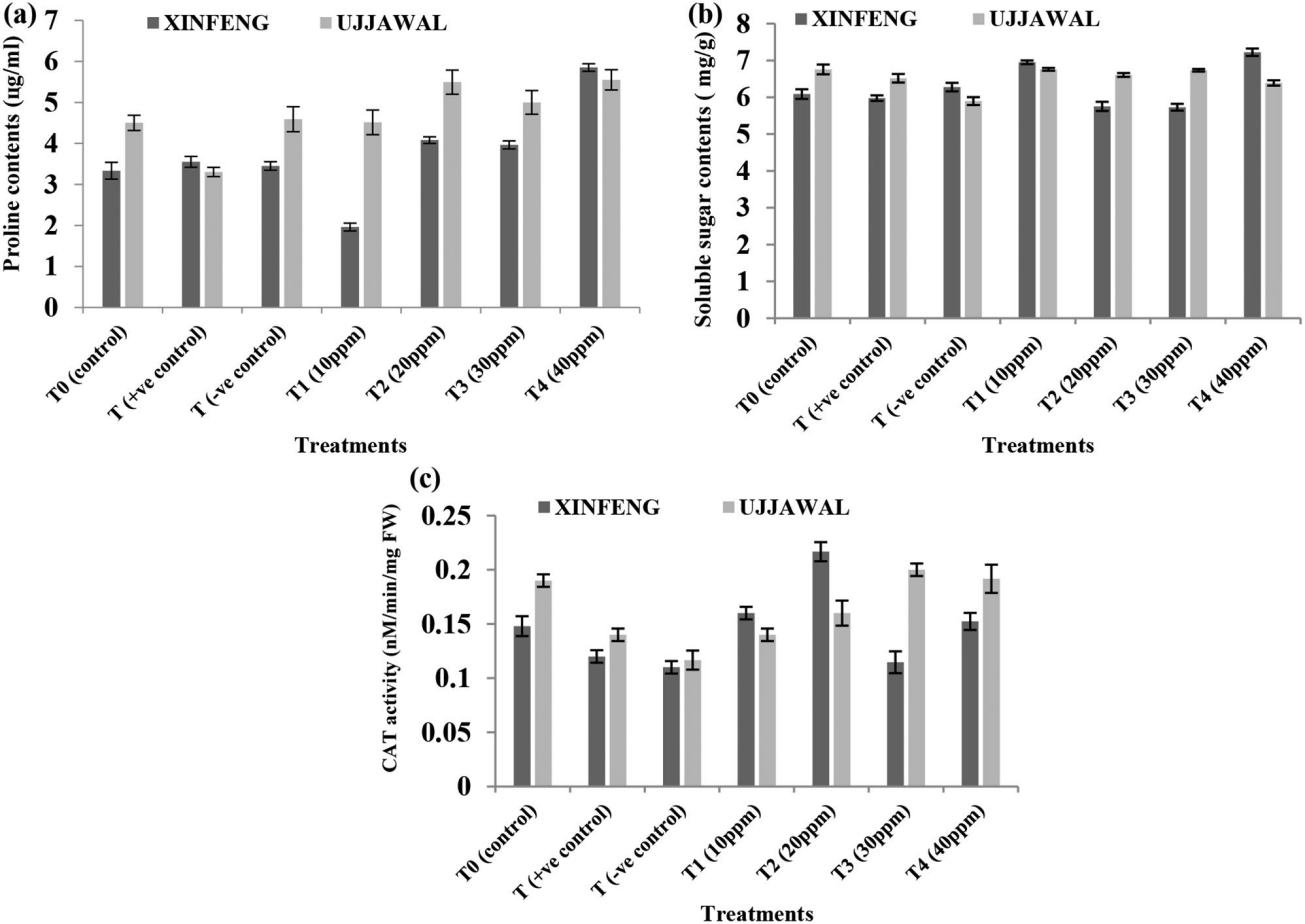
Variation in biochemical parameters of two tomato varieties in response of various treatments of green fabricated AgNPs

Metabolic alterations in response of BC disease infestation and mitigation by green fabricated AgNPs in tomato plants were evaluated in terms of stress responsive non-enzymatic (proline and soluble sugar content) and enzymatic (catalase) activities. It was observed in the present study that level of proline and soluble sugar contents (osmoprotectant) was relatively increased in response to *Cmm* inoculation induced biotic stress as compared to healthy plants (T0) in both tomato varieties. Moreover, highest values for proline contents were recorded in response of foliar application of 40 ppm solution of AgNPs in both varieties among all the treatments (Fig. 5a and b). Significant variation was observed in enzymatic activity of CAT in response to bacterial canker disease infestation and its mitigation by various treatments of green fabricated AgNPs. Activity of CAT was significantly low in infected plants as compared to healthy one. On the other hand, maximum increase in CAT activity in both diseased and healthy plants was observed in response of foliar application of 20 ppm solution of AgNPs among all treatments (Fig. 5c).

### Disease Severity

**Fig. 6:**
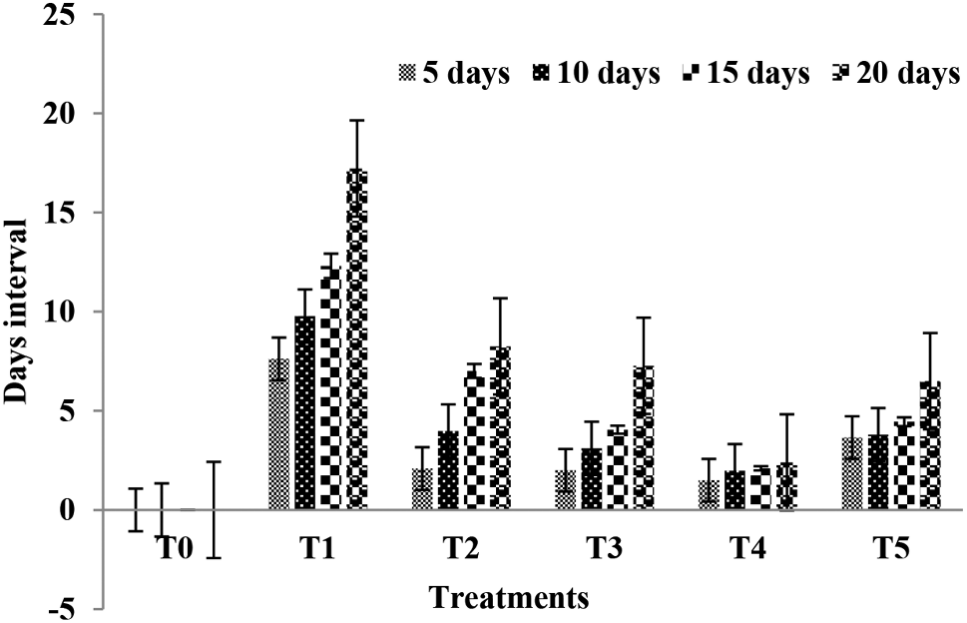
Assessment of disease incidence at different concentrations of AgNPs against bacterial diseases at different day intervals. Where 0 = free from infestation, T1 = 25% infested leaf area; T2 = 50% leaf area infested; T3 = 75% leaf area infested; T4 = 100% leaf area infested

The occurrence of bacterial canker disease was gradually reduced with the passageway of time in entirely the usages of green fabricated AgNPs capable of 20 days. Under biotic stress, the tomato plants which were not smoked with the green fabricated AgNPs s exposed maximum septicity index integrities at various day intervals shown in Fig. 6. The exogenously 30 ppm concentration of the green fabricated AgNPs sprayed on tomato plants recorded through reduce disease severity. The septicity index standards in response to 10, 20, 30, and 40 ppm concentration of *M. oleifera* leaves extract-mediated AgNPs were suggestively decreased after 20 days while distinct with T1 treatment. After 20 days in all the useful treatments, disease severity was enhanced.

## Discussion

Leaf extract of *M. oleifera* showed potential to reduce and cap the silver ions to AgNPs in aqueous solution which suggest that leaf extract contain various bioactive phytocompounds act as reducing and stabilizing agent for the synthesis of nanoparticles. It was also confirmed in present study by detection of various functional groups such as esters, alcohols, ethers, carbonyl compounds, alkanes, alkenes, alkyl halides, amines, aldehydes and aromatic compounds in FT-IR spectrum (Fig. 2b). Additionally, these bioactive compounds not only involved in the green fabrication of nanoparticles they also boost the antioxidant defence mechanism of plant to mitigate the oxidative damages [26]. Aromatic compounds such as flavonoids, phenolics and terpenoids are the key active constituents in the leaf extract of *M. olifera* known for capping of metal ions and reduction of metal salts into nanoparticles [27–29]. Nevertheless, it is still unclear which active plant compounds are involved in the biosynthesis of metal nanoparticles. Therefore, secondary metabolites and a set of plant compounds were considered active constituents in the present study. Silver nanoparticles also exhibited surface plasmon resonance (SPR) peaks in the range of 350 to 400 nm that confirms the precipitation of AgNPs in aqueous state. These findings are also in line with earlier evaluations of AgNPs synthesis and characterizations [30–32]. Furthermore, EDX spectrum and XRD diffraction pattern of metallic silver ions in the green fabricated AgNPs are also confirmatory with the findings of previous studies, who also reported the antimicrobial activity of silver nanoparticles [33–35]. Scanning electron microscopic (SEM) images of rectangular fragments *of M. oleifera* leaf confirmed the synthesis of extract mediated AgNP as previously reported by [36].

Present study evaluated the antibacterial latency of substantially made AgNPs against BCT triggered by *Cmm* in tomato as it was reported previously in other crops [37]. Foliar application of *Moringa* plant extract with various concentrations of green fabricated AgNPs improved the morphological growth measured in terms of shoot length (cm), tomato plant weight (g), plant fresh biomass (g), root length (cm), root dry weight (g), root fresh weight and plant dry biomass (g), by supressing the pathogenicity of *Cmm*. At different day intervals, 30 ppm of AgNPs was found to be most effective by reducing the severity of the disease in comparison to all other concentrations. These results are in line with previous studies reported that foliar treatment of various concentrations of AgNPs inmroved the development of wheat, corn and bean crops [38–41]. It is inferred that increase in the length and weight of plant parts (shoot and root) in response to foliar application AgNPs is maybe linked with the bioactive potential of green fabricated nanomaterial which stimulate the physiological process like cell division, elongation and differentiation as well as enhanced nutrient uptake and their translocations that leads to biomass production [42, 43]. Previous study also reported that plant growth promotion in response of AgNPs is also linked with increased level of endogenous growth regulators such as gibberellin, auxin and zeatin that initiate the regular pattern of cell division [44, 45]. Penetration and accumulation AgNPs in the plant tissues also triggers the gene expression responsive for the biosynthesis of abscisic acid and inhibition of ethylene production which leads to growth and development of healthy roots [46]. Furthermore, growth stimulation responses were high at low concentration and inhibited at higher concentration in the present study may be attributed to mitotic index inhibition and spindle alteration in meristemtic cells as reported by Fouad and Hafez in *Allium cepa* [47].

It can also hypothesize from the findings the increase in fresh plant biomass is maybe due to increase in relative water content, chlorophyll content which was significantly increased in both varieties of tomato in response of AgNPs under biotic stress conditions. It was reported in earlier studies that biotic stress reduces the physiological activities such as rate of photosynthesis, turgor pressure and nutrient uptake but AgNPs works antagonistically [48, 49]. Singh et al. [50] also reported a significant increase in physiological parameters in response of AgNPs as they trigger the production of osmoprotectants, and stress responsive antioxidants. Similar findings were reported in wheat plants by Satti et al. [51] who suggested that AgNPs inhibit the microbial growth by inhibiting their DNA replication and protein degradation and enzyme inactivation, ATP deprivation that hampers the overproduction of compounds responsible for cellular damages in the host plant. Blum [52] reported that accumulation of organic solutes in response of AgNPs stabilizes a low water potential by increasing the relative water content in the cells and enhances the membrane stability by reducing the lipid denaturation under stress conditions. It was confirmed in a previous study suggested that exogenous application of AgNPs lowered the MDA content in tobacco plants and electrolyte leakage by improved cell membrane stability [53].

In present study, photosynthetic pigments (chlorophyll a, chlorophyll b and total chlorophyll) were increased in response of various concentrations of AgNPs suggest that photosynthetic activity was improved that enhanced the plant growth in both verities of tomato. It also suggest that AgNPs treated plant absorbed the sufficient nitrogen and magnesium and assembled them in photosynthetic pigment i.e. chlorophyll [54]. Similar findings were reported in wheat and maize plants treated with different levels of biogenic AgNPs coated with *M. olifera* extract [39, 55, 56]. Additionally, it was also reported that chlorophyll content in maize plants increased in response to small concentrations (10–50 µl/l) of AgNPs but decreased in response to higher concentrations. It suggests that optimal range of metal nanomaterials positively regulates the physiological processes and plant growth while high concentration causes potential toxicity of metal ion that disrupts the cellular activities and induces the oxidative stress [57, 58]. Proline is non-enzymatic antioxidant and known to alleviate the negative effects of biotic and abiotic stresses by scavenging the reactive oxygen species and inhibit the metal chelator’s activity [59]. In present study, proline and soluble content was increased in response of all treatments of green fabricated AgNPs as it was earlier reported in tomato plants treated with both chemically and biologically synthesized AgNPs [60]. Proline enhances the stress tolerance in plant as it potentially acts as apoptosis inhibitor [61] and soluble sugars maintain the osmotic adjustments and assist the detoxification of ROS [62]. Mishra & Bäuerle [63] and Ahmed et al. [64–66] reported similar findings in response of nanomaterials in food crops such as wheat, pea. Furthermore, our findings are in consistent with findings of Mehrin et al. [67], who reported variation in amino acid contents in tomato plants treated with AgNPs as compared to untreated plants. The activity of CAT (stress responsive antioxidant enzyme) increased in the response of Ag and AgNPs as reported in a recent studies who reported that activities of enzymes were high with high concentration of AgNPs in *S. lycopersiucm*, *Oryza sativa* and *Lemna gibba* plants [68–71]. Earlier it was reported that microbial stresses induce the overproduction of ROS and AgNPs trigger the production of stress responsive enzymes such as CAT, peroxidase, superoxide dismutase that scavenge them and improve the metabolic processes and plant growth [72].

**Fig. 7:**
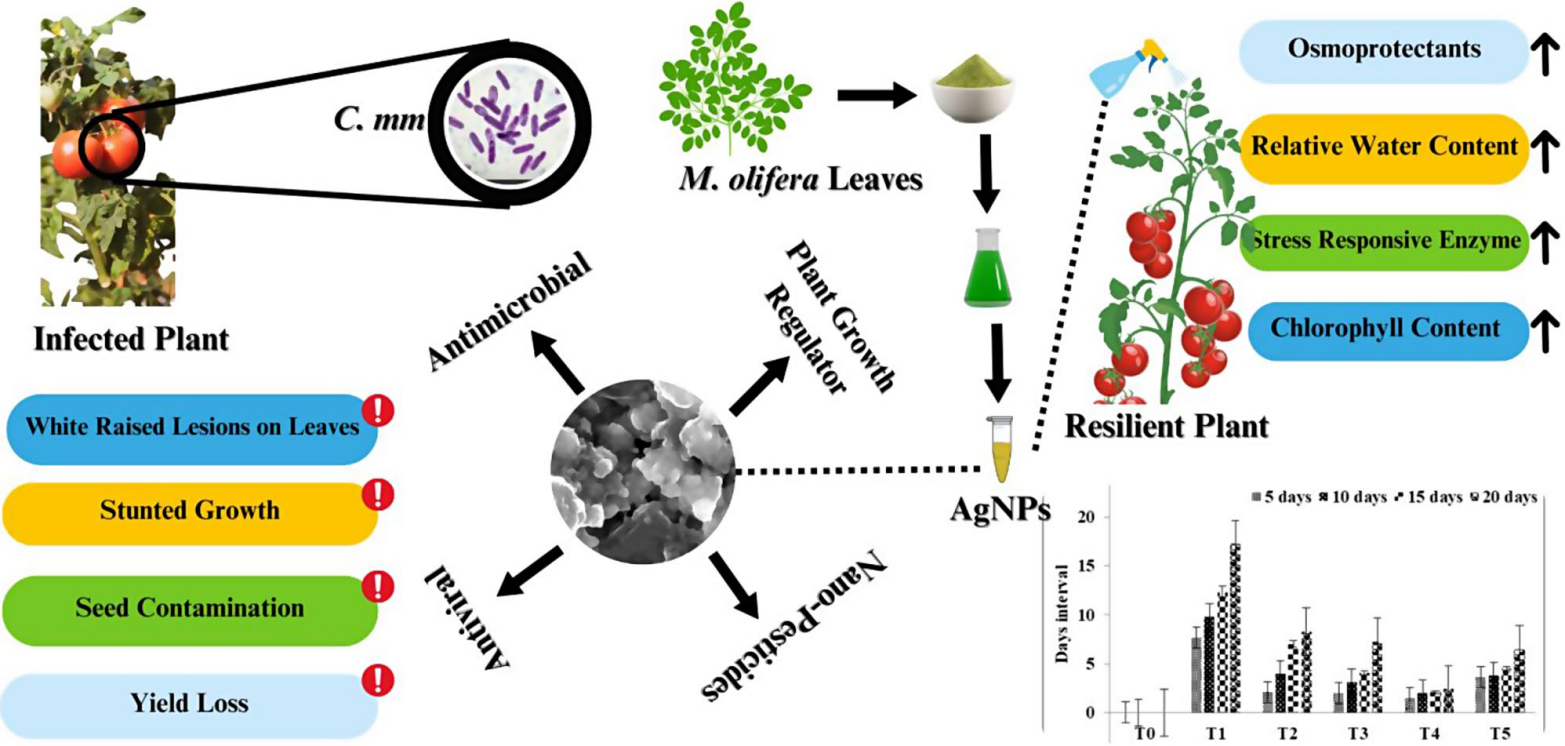
A schematic model for AgNPs induced mitigation of bacterial canker disease in tomato plants

AgNPs also exhibited greater antibacterial potential than bacteriostatic potential which is in accordance with the findings of a previous study [73]. It suggests that green fabricated AgNPs can easily eradicate the pathogenic microbes rather than simply impeding the extent of contamination as reported by Kumari et al. [74]. , . Additionally, it was observed in prrsent study that antimicrobial potential of AgNPs not only effective for seed germination stages also improves the growth at subsequent growth stages in tomato plants till harvest. Several previous studies measured a critical value of AgNP toxicity from concentration ranging from 5 to 1000 ppm, while 10 ppm, 20 ppm, 30 ppm and 40 ppm were often found very effective under different conditions [75]. We showed that the toxicity of AgNPs is influenced by the concentration utilized; for example, this evaluation revealed no important lethal impacts on the propagation or sprouting of roots of tomato plants. A proposed simple schematic model for the AgNPs induced stress management against bacterial canker disease in tomato plants is given in the Fig. 7.

There might be two possible causes: (i) the accumulation of AgNPs after they are exposed to the environment loses their toxicity [76], and (ii) the ability of the endosperm and germ/seed coat to assist in the replacement of a filter, transient water and capture of metals [77]. Physico-chemical properties of AgNPs altered under open field conditions due to several chemical reactions dissolution, oxidation, and agglomeration that affect the surface and reactivity and lower the harmful impacts [78]. Photodegradation and biodegradation may also breakdown AgNPs into less toxic forms or maybe these particles get adsorbed and complexed with organic matter and or other minerals [79]. However, the particular bactericidal application of Ag^+^ has not yet been elucidated because Ag^+^ is usually recognized for its ability to be discharge [80, 81]. There is a rough relationship between the apparent zone of the AgNPs and the concentration of loose Ag^+^. AgNPs in the maximum surface zone will discharge the maximum amount of Ag^+^ and vice versa. The biosynthesized AgNPs suppressed the incidence of tomato disease (bacterial canker) as compared to control trial under glasshouse conditions. The plant growth parameters were greater in the greenhouse than in the control. The results suggested that the AgNPs fabricated by *M. oleifera* could be an optimal phase for protecting plants against the BC disease triggered by *Cmm*. This study concluded that a 30 ppm concentration of AgNPs decreased the severity index in contrast to canker on different days. The present results are similar to those of [33], who described the influence of biosynthesized AgNPs on brown spot infection in Kinnow mandarin. Our findings are also in strong line with those of [82], who documented the occurrence of disease on the Kinnow mandarin in various areas affected by *X. axonopodispv. citri*. The production of NADPH oxidase, peroxidases and amine oxidases in the cell is enhanced by ROS under biotic stress [83].

## Conclusion

*M. oleifera* have tremendous therapeutic and nutritional value and also exhibited strong potential for reduction and capping of silver ions in silver-nanomaterials from silver nitrate in aqueous solution. In present study, green fabricated AgNPs exhibited antimicrobial potential and suggest that these nanomaterials can be used to manage the microbial diseases. Present study evaluated the effect of various concentrations of AgNPs to manage the bacterial canker disease in tomato caused by *Clavibacter michiganensis* subsp. *Michiganensis*. It has been concluded that foliar application of low concentration of AgNPs is more effective to enhance the growth and production in both healthy and diseased plants while high concentration can induce toxic effects in plants that ultimately hinders the growth. This is the first report of utilizing green fabricated AgNps with leaf extract of M. olifera against bacterial canker disease in tomato caused by *Cmm*.

## Data Availability

All relevant raw data, will be freely available on request.
